# Paradoxical Thromboembolism/ST-Elevation Myocardial Infarction via a Patent Foramen Ovale in Sub-Massive Pulmonary Embolism Following an Upper Extremity Deep Venous Thrombosis: Is It Time for a Change in the Standard of Care?

**DOI:** 10.14740/cr335w

**Published:** 2014-07-20

**Authors:** Deepali Nivas Tukaye, Rodrigo Silva Cavallazzi

**Affiliations:** a Division of Cardiology, The Ohio State University, Columbus, OH 43210, USA; b Division of Medicine Pulmonary Critical Care, University of Louisville, Louisville, KY 40202, USA

**Keywords:** Paradoxical thromboembolism, Upper extremity DVT, STEMI, Sub-massive pulmonary embolism

## Abstract

The objective of this case study is to discuss a rare case of proven paradoxical thromboembolism captured in-transit. A 23-year-old female with a diagnosis of right internal jugular deep vein thrombus who developed acute onset chest pain, dyspnea and hypotension, was selected for the study. Sub-massive PE and STEMI were diagnosed. Transthoracic echocardiogram revealed a left ventricular (LV) mass moving across the aortic valve. Soon after, the patient developed numbness of right extremities with non-palpable pulses. A transesophageal echocardiogram revealed absent LV mass, PFO, left atrial mass entering through the PFO and emboli in bilateral pulmonary arteries. We report a case of sub-massive PE and paradoxical proven coronary and upper extremity embolism, captured in-transit, following destabilization of an UEDVT in a patient with PFO.

## Introduction

Upper extremity deep venous thrombosis (UEDVT) accounts for approximately 5-10% [1] of all cases of DVT (incidence: 0.4/10,000) [1] with lower risk of embolic complications than lower extremity DVT (LEDVT) [2]. UEDVT generally afflicts young, healthy individuals resulting in long-term morbidity affecting the quality of life [3]. Paradoxical embolism in association with patent foramen ovale (PFO) is well documented commonly giving rise to embolic stroke or extremity vaso-occlusion [4]. Paradoxical coronary embolism is a rare phenomenon with few cases reported in literature.

## Case Report

**Figure 1:**
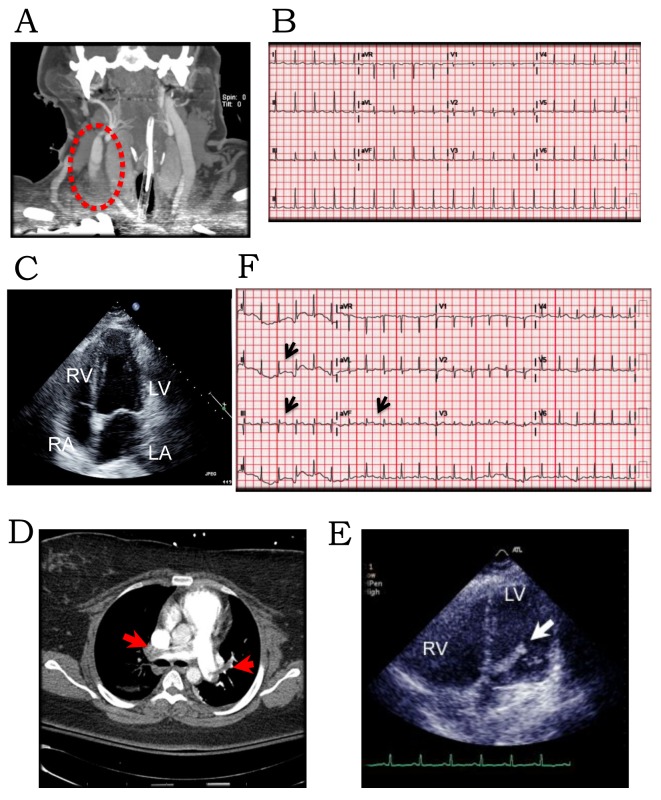
(A) CT angiogram of head and neck demonstrating the right internal jugular vein thrombus. (B) Baseline EKG in normal sinus rhythm no ST-T changes. (C) Transthoracic ECHO 4-chamber view of the heart with no thrombus during first hospitalization. (D) CT PE protocol revealing emboli in right and left PAs (arrows). (E) Transthoracic ECHO 4-chamber view of the heart revealing thrombus in the LV cavity extending across the mitral valve with point of origin in the LA (arrow). (F) EKG demonstrating ST elevation in II, III and aVF.

**Figure 2:**
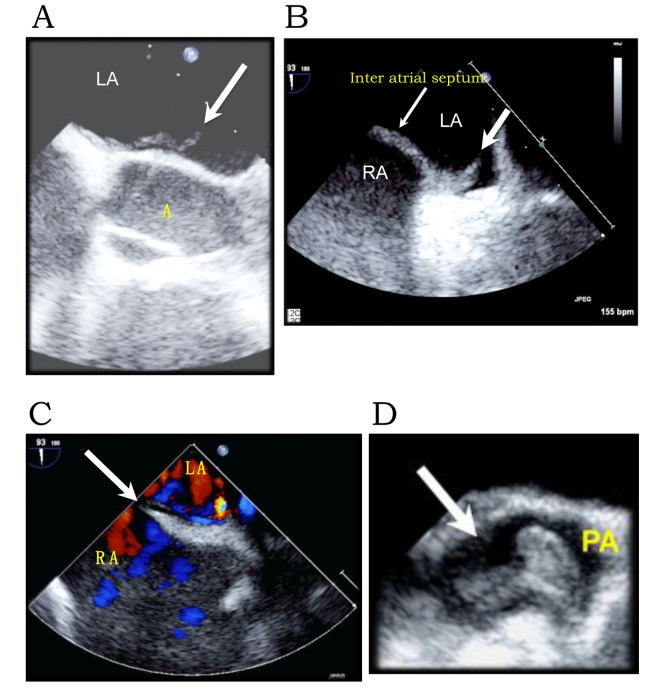
Transesophageal ECHO. (A) Serpiginous thrombus in the LA. (B) Thrombus straddling across the PFO (broad arrow). (C) Color Doppler demonstrating flow across the inter-atrial septum via the PFO (broad arrow). (D) Embolus in the PA (arrow).

**Figure 3:**
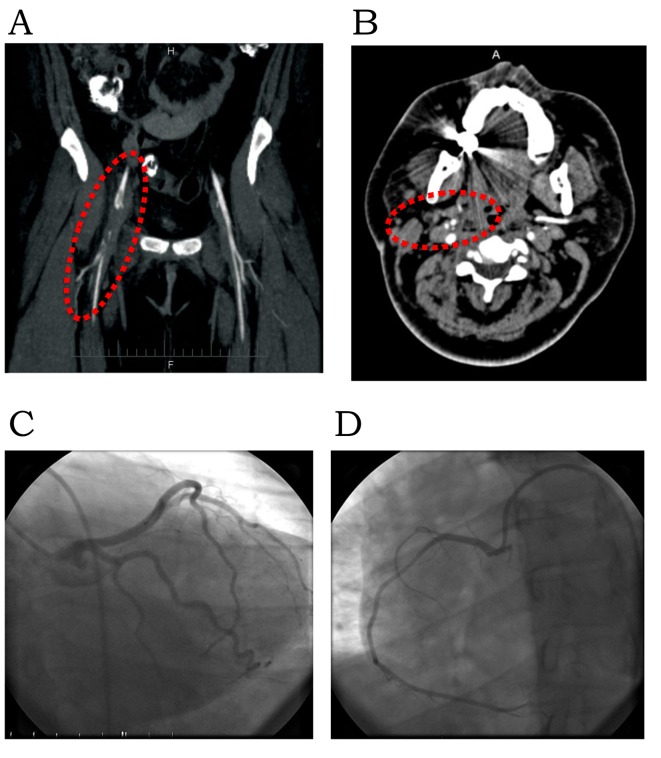
Paradoxical thromboembolism. (A) CT angiogram of lower extremities with emboli (filling defects; dotted circle) in the right illio-femoral arteries. (B) CT angiogram of upper extremities with emboli (filling defects; dotted circle) in the right subclavian artery. Cardiac catheterization, (C) left coronary artery; (D) right coronary artery.

A 23-year-old African-American female with a history of right internal jugular vein thrombosis (Fig. 1A) following a gunshot wound to the neck in the prior month, was readmitted for her tracheostomy revision. Her initial course was uneventful with baseline laboratory tests, EKG (Fig. 1B) and vital signs within normal limits. On day 3, she developed shortness of breath and chest pain. EKG showed dynamic ST-elevation in II, III, aVF (Fig. 1F) and elevation of cardiac enzymes with peak troponin of 20.6 (peak: CKMB = 39, CK = 839, CKMB index = 7) consistent with myocardial infarction. She was diagnosed with ST-elevation myocardial infarction (STEMI) and started on acute coronary syndrome protocol. Secondary to associated hypoxia and respiratory distress she was placed on mechanical ventilation. There was no clinical evidence of heart failure or pulmonary edema on clinical evaluation; hence, pulmonary embolism (PE) was suspected. Chest CTA demonstrated bilateral pulmonary artery emboli (Fig. 1D). A transthoracic echocardiogram revealed global left ventricular (LV) hypokinesis and an LV mass plopping across the aortic valve during systole (Fig. 1E). There was associated dilatation of right atrium (RA) and ventricle with significant tricuspid regurgitation (systolic pulmonary artery (PA) pressure, 68 mm Hg). She was diagnosed with sub-massive PE. On her way to the cardiac catheterization laboratory, given evidence of STEMI, she complained of numbness and paresthesia in her right extremities. The right radial, brachial, posterior tibial, popliteal and dorsalis pedis pulses were no longer palpable on physical exam but dopplerable. Her right extremities were cold to touch. Emergent cardiac catheterization demonstrated clean coronaries, ruling out atherosclerotic etiology, and right illio-femoral emboli. Given the recent history of right internal jugular vein DVT, the LV mass was deemed to be a thrombus clinically. Thrombolytics were withheld given the high risk for further systemic thromboembolism/stroke from lysis of the LV thrombus. A transesophageal echocardiogram revealed normal LV cavity with a serpiginous thrombus (Fig. 2A), a significant PFO (Fig. 2C), a tubular mass in the RA straddling across the PFO into left atrium (LA) (Fig. 2B) and non-obstructive embolus in the PA (Fig. 2D). At this time, CTA of the extremities revealed non-occlusive emboli in the right illio-femoral (Fig. 3A), brachiocepalic (Fig. 3B), subclavian (Fig. 3B), brachial and radial arteries. Embolectomy was deferred, as extremity perfusion was not compromised and the patient was clinically unstable. Compression Doppler ultrasound (U/S) of right extremities demonstrated no lower extremity and no subclavian and axillary vein DVT. Based on preceding events we hypothesize that the right internal jugular vein thrombus had destabilized causing the sub-massive PE and the elevated right atrial pressures had facilitated paradoxical embolism via the PFO causing transient STEMI and peripheral arterial thromboembolism. The above events transpired over a period of 6 h and have been presented chronologically.

Cardiothoracic surgery was consulted for removal of the LV mass. However, the rapid succession of events and hemodynamic instability precluded surgery. She remained afebrile and blood cultures were negative. She was managed with anticoagulation and supportive care. Initial hypercoagulability workup was weakly positive for lupus anticoagulant (positive direct Russell viper venom test, negative anti-B2-glycoprotein). She was discharged on day 25 on anticoagulation with warfarin, physical therapy and regular follow-up.

### Follow-up

She showed moderate improvement in her mobility and exercise-tolerance with physical therapy. Compression upper extremity Doppler at 3 months was negative for right internal jugular vein DVT. A 6-month transthoracic echocardiogram was unremarkable. At 1-year follow-up her anticoagulation was discontinued after a repeat transesophageal echocardiogram was negative for an intracardiac thrombus. Also, at 1-year UE Doppler was negative for UEDVT. Lupus anticoagulant at 12 weeks off anticoagulation was negative. She continues to have a tracheostomy due to difficulties in downsizing.

## Discussion

PE complicates about 6% cases of UEDVT as compared to 15-32% cases of LEDVT [1]. Paradoxical thromboembolism from underlying PFO is a dreaded complication of DVT. Proven and cryptogenic cerebrovascular accidents from paradoxical thromboembolism are well documented in literature. Cohnheim first described paradoxical embolism stemming from a PFO in 1877. Paradoxical coronary embolism secondary to DVT is a relatively rare phenomenon with < 50 cases of proven [5-9] and presumed [9-21] cases reported in literature based on our literature search. Paradoxical thromboembolism is proposed to have occurred if the following criteria are fulfilled [14]: 1) evidence of arterial emboli in the absence of source in the left heart; 2) identified source in the venous system; and 3) presence of a communication between the venous and arterial circulation. The diagnosis is defined as “proven” when an embolus is identified in the abnormal communication between the venous and arterial systems. Most cases described in literature are presumed with no evidence of actual transit of thrombus across a left-right intracardiac shunt. There are very few documented cases of proven paradoxical coronary thromboembolism. To best of our knowledge, this is the first case of proven paradoxical coronary embolism resulting from an UEDVT along with sub-massive PE and extremity embolism. We have also been able to capture the transit of the embolus using multiple imaging modalities.

Most UEDVTs tend to occur in the subclavian-axillary vein segments [22]. Our patient developed a right internal jugular DVT. Her history was negative for sedentary lifestyle, previous DVT, smoking, oral contraception and personal/family history of hypercoaguability. Her hypercoaguability workup was negative. We hypothesize that the clot may have been related to trauma secondary to gunshot wound to the neck.

The likely course of events was destabilization of the right internal jugular DVT leading to sub-massive PE. Raised right atrial pressures from the PE resulted in opening up of the PFO with transit of thrombus from RA to LA. The thrombus most likely propagated and then fragmented to produce the systemic thromboembolism. The diagnosis of STEMI was made based on EKG, cardiac enzymes and echocardiogram (ECHO) findings. Given clean coronaries and visualization of thrombus across the PFO, the STEMI was deemed to be due to transient paradoxical embolism. One could argue that the ECG and cardiac enzyme changes were related to the PE. However, the inferolateral ECG changes in absence of changes in V1-3 and significant elevation in cardiac enzymes are inconsistent with PE alone. Fortunately, after the initial rapid fragmentation and embolization, there were no further embolic events.

Incidence of PFO in general population ranges from 24% to 27% [23]. Incidence decreases with age [24]. In studies evaluating cryptogenic strokes, the incidence of PFO has been estimated to be around 40-50% in individuals < 55 years [24]. Based on very limited clinical and autopsy studies, coronary thromboembolism appears to account for 5-10% cases of all paradoxical thromboembolism [25]. This number may be an underestimate given the diagnosis is more likely to be missed than not. Studies have also shown that individuals suffering from paradoxical thromboembolism tend to be younger, leaner and less likely to have thrombophilias [3]. In the last decade there has been an exponential increase in intravascular catheter-based procedures undertaken in young individuals with a corresponding rise in the incidence of DVT, especially UEDVT. The consequences of thromboembolic events in otherwise healthy young individuals are catastrophic and severely compromise quality of life. Based on this, we believe that it is time to reevaluate how we manage DVT in otherwise young healthy individuals. Currently, anticoagulation for extended periods of time is the standard of care for management of DVT. However, it may be prudent to evaluate for significant PFO in young healthy individuals with DVT. Large scale studies are needed to determine if including surveillance for significant PFO and the use of localized thrombolytic therapy/embolectomy in the management of DVT will improve outcomes in young and otherwise healthy patients with provoked DVT.

In conclusion, embolic complications in UEDVT are rare but in combination with a PFO they can be a source of significant morbidity and mortality. Even though current guidelines recommend anticoagulation only as management of UEDVT, we propose that young individuals with UEDVT be screened for PFO and that DVT be managed more aggressively with thrombolysis or embolectomy.

## Abbreviations

RA: right atrium
LA: left atrium
RV: right ventricle
LV: left ventricle
PA: pulmonary artery
A: aorta
